# Perspectives and potential approaches for targeting neuropilin 1 in SARS-CoV-2 infection

**DOI:** 10.1186/s10020-021-00423-y

**Published:** 2021-12-27

**Authors:** Svetlana P. Chapoval, Achsah D. Keegan

**Affiliations:** 1 Department of Microbiology and Immunology, University of Maryland School of Medicine, Baltimore, MD USA; 2 Center for Vascular and Inflammatory Diseases, University of Maryland School of Medicine, 800 West Baltimore Street, Baltimore, MD 21201 USA; 3 Program in Oncology at the Greenebaum Cancer Center, University of Maryland School of Medicine, Baltimore, MD USA; 4 SemaPlex LLC, Ellicott City, MD USA; 5 VA Maryland Health Care System, Baltimore VA Medical Center, Baltimore, MD USA

**Keywords:** ACE2, angiotensin converting enzyme 2, NRP-1, neuropilin-1, Molecular structures, SARS-CoV-2, severe acute respiratory syndrome coronavirus 2, Spike protein, COVID-19, coronavirus disease 2019, Host immune response, Immunotargets and strategies, Comorbidities, CendR C-end rule, RBD, receptor-biding domain

## Abstract

Severe acute respiratory syndrome coronavirus 2 (SARS-CoV-2) is a novel type b coronavirus responsible for the COVID-19 pandemic. With over 224 million confirmed infections with this virus and more than 4.6 million people dead because of it, it is critically important to define the immunological processes occurring in the human response to this virus and pathogenetic mechanisms of its deadly manifestation. This perspective focuses on the contribution of the recently discovered interaction of SARS-CoV-2 Spike protein with neuropilin 1 (NRP1) receptor, NRP1 as a virus entry receptor for SARS-CoV-2, its role in different physiologic and pathologic conditions, and the potential to target the Spike–NRP1 interaction to combat virus infectivity and severe disease manifestations.

## Background

SARS-CoV-2 infected over 224 million people worldwide leading to more than 4.6 million deaths. Currently, there is no FDA-approved treatment for the SASR-CoV-2 viral disease. Therefore, there is an urgent need for anti-viral therapeutics to treat this and future SARS infections. It is now well-established that SARS-CoV-2 entry into cells is initiated by its Spike protein priming by the transmembrane protease serine 2 (TMPRSS2) and binding to its main receptor in human tissues, the angiotensin‑converting enzyme 2 (ACE‑2) (Hoffmann et al. 2020b; Wang et al. 2020b; Yan et al. 2020). However, recent data suggest that there are additional mediators of viral entry and cell infectivity (Cantuti-Castelvetri et al. 2020; Daly et al. 2020; Radzikowska et al. 2020; Root-Bernstein 2021). One such mediator is neuropilin 1 (NRP1) as reported in several recent preclinical and clinical studies (Cantuti-Castelvetri et al. 2020; Daly et al. 2020; Davies et al. 2020; McFarland et al. 2021; Moutal et al. 2021). In this review, we explored the details of NRP1 expression and function in health and diseases and perspectives of its targeting in order to prevent or abrogate a severe SARS-CoV-2 infection.

## SARS-CoV-2 structure

Several recent publications provide detailed descriptions of SARS-CoV-2 structure and its unique features distinguishing it from SARS-CoV or MERS-CoV (Chen et al. 2020; Chuckran et al. 2020; Hoffmann et al. 2020a, b; Finkelstein et al. 2021; Kadam et al. 2021; Letko et al. 2020; Papageorgiou and Mohsin 2020; Sternberg and Naujokat 2020; Walls et al. 2020; Wang et al. 2020a; Witkowska 2020). SARS-CoV-2 is a single-stranded positive sense RNA virus. Its membrane is composed of several structural proteins such as membrane (M), spike (S), and envelope (E) proteins. Viral RNA bound to helical nucleocapsid phosphoproteins (N) is positioned inside the virion. The spike protein of SARS-CoV-2 is an attractive antiviral target and main component (in form of mRNA or DNA) of all FDA- and EU CHFS-approved anti-COVID-19 vaccines (Chen et al. 2020; Letko et al. 2020; Wang et al. 2020a). Spike is a transmembrane homotrimeric glycoprotein of ~ 180 kDa that belongs to the class I of trimeric fusion proteins and consists of two subunits, S1 and S2 (Wang et al. 2020a). The S protein is cleaved by the transmembrane protease serine 2 (TMPRSS2) which is preferentially expressed on epithelial cells in the airways such as alveolar epithelial type II cells (ATII cells) (reviewed in Sternberg and Naujokat 2020; Hoffmann et al. 2020b). The TMPRSS2-mediated S protein cleavage and priming are required for its binding to ACE2 receptor, membrane fusion, and cell entry (Sternberg and Naujokat 2020). The S1 subunit of S protein contains a receptor-binding domain (RBD). The S2 subunit contains several domains including a fusion peptide for fusion of virus with a host cell membrane. The S1/S2 boundary includes the cleavage site for the subtilisin-like host cell protease furin which is expressed in all human tissues (Hoffmann et al. 2020a; Sternberg and Naujokat 2020; Walls et al. 2020; Wrapp et al. 2020). The furin cleavage site of S protein is believed to contribute to the high virulence and tissue tropism of SARS-CoV-2 in humans because of ubiquitous expression of furin and furin-like proteases (Hoffmann et al. 2020a; Walls et al. 2020; Wrapp et al. 2020). Moreover, the envelope proteins of several other viruses such as HIV, influenza, dengue fever, Ebola virus, and Marburg virus utilize furin or furin-like proteases for their cleavage and virus activation (Shiryaev et al. 2007).

## ACE2 structure and function

Angiotensin-converting enzyme 2 (ACE2) is the main cell surface receptor for SARS-CoV-2 (Hoffmann et al. 2020b; Wang et al. 2020b; Yan et al. 2020). ACE2 functions as a physiological counter-balance molecule of ACE. It is a type I integral membrane carboxypeptidase which structure has been solved (Turner 2015; Wan et al. 2020). ACE cleaves vasodilating angiotensin 1 into angiotensin 2 which displays a vasoconstricting function (Hamming et al. 2004; Turner 2015). In contrast, ACE2 causes vasodilation by cleaving and hydrolyzing angiotensin 2 into angiotensin decapeptide 1–7 which signals through the G-protein coupled MasR to induce Akt phosphorylation and NOS production (Bader 2013). Structural biology techniques such as Cryo-Electron Microscopy (Cryo-EM) and X-ray crystallography techniques detail the Spike protein interactions with ACE2 guiding the ongoing therapeutic and vaccination efforts (reviewed in Papageorgiou and Mohsin 2020; Chekol Abebe et al. 2021). Rather low to absent ACE2 expression was detected in all compartments of human respiratory tract. The analysis of three distinct scRNAseq datasets demonstrated low ACE2 expression in ATII cells, bronchial, goblet, and ciliated cells within the airways (Hikmet et al. 2020). Other recent studies reported high ACE2 and transmembrane serine protease 2 (TMPRSS2) expression in nasal epithelium and in lung parenchyma (Sungnak et al. 2020; Yang et al. 2020). Interestingly, analysis of the ACE2 and TMPRSS2 expression pattern in individual cells in the lung and in subsegmental bronchial branches by scRNAseq demonstrated a strong TMPRSS2 expression in both tissues whereas ACE2 was predominantly detected in a transient secretory cell type (Lukassen et al. 2020). The transcriptome analysis also showed that ATII cells co-expressed ACE2 with TMPRSS2. No sex-, age-, or gender-related differences were observed in ACE2 expression in individual cell types in lung cells or in the subsegmental bronchial lung tissue. However, the cell surface receptor expression was not examined in this study. These single cell transcriptome data led to several explanations for the observed relatively high human-to-human transmission of SARS-CoV-2 when compared to SARS-CoV or MERS-CoV, namely: (a) the binding of SARSCoV-2 to another, yet unknown receptor on the host cell surface, (b) enhanced cleavage of the SARS-CoV-2 S protein resulting in higher efficiency of the virus’ entry into the cell, and (c) additional host factors increasing the virus entry into the cell by facilitating membrane fusion. All these can explain why SARS-CoV-2 readily infected tissues with relatively low or absent ACE2 expression, such as the respiratory tract and nervous system, pointing to other potentially important factors/receptors for virus entry (reviewed in Chekol Abebe et al. 2021).

## NRP1 structure and function

**Fig. 1:**
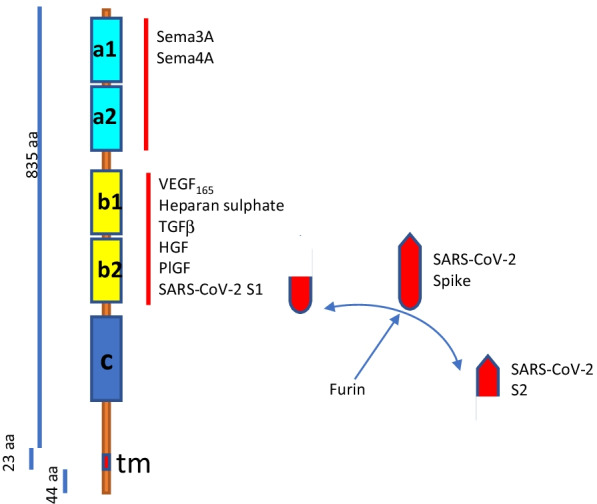
Neuropilin 1 structure and ligands. NRP1 consists of a large 835 aa extracellular domain, short transmembrane (23 aa) domain, and cytoplasmic (44 aa) portion. Semaphorins 3A and 4A bind to the a1/a2 domain (also termed CUB domain due to its homology to complement proteins C1r and C1s), whereas VEGF_165_, heparan sulphate, TGFb, hepatocytes growth factor (HGF), placental growth factor (PlGF), and SARS-CoV-2 Spike S1 protein bind to the b1/b2 domain (also termed as a coagulation factor homology domain). The a and b domains are critical for binding to the corresponding ligand, whereas the c domain (mephrin or MAM) is important for receptor homodimerization and heterodimerization with Plexin family members. Tm-transmembrane domain. The S1/S2 boundary of the SARS-CoV-2 Spike protein includes the cleavage site for the subtilisin-like host cell protease furin which is expressed in all human tissues. Cleaved S1 through its C-end rule or CendR motif (C-terminal basic sequence motif) directly binds to the b1/b2 domain of NRP1. This binding promotes virus entry and infection

Two recent reports in *Science* demonstrated that the SARS-CoV-2 Spike protein can also bind to the b1b2 domain of NRP1 (Cantuti-Castelvetri et al. 2020; Daly et al. 2020). Unlike SARS-CoV, SARS-CoV-2 contains a polybasic amino acid sequence (^682^RRAR^685^) which serves as furin cleavage site which, when cleaved, directly binds NRP1 (Fig. 1) significantly potentiating viral entry and increasing the in vitro cell infectivity with WT virus by 40–70% depending on the cell type used in the experiments as shown by blocking of NRP1 with Ab or shRNA (Cantuti-Castelvetri et al. 2020; Daly et al. 2020). NRP1 is a single-pass transmembrane glycoprotein, its extracellular portion is involved in semaphorin 3A (Sema3A) and VEGF_165_ binding (Kolodkin et al. 1997; Chapoval et al. 2009; Wild et al. 2012) whereas the c-domain and transmembrane portion are involved in receptor dimerization and heteromerization (Wild et al. 2012) (Fig. 1). NRP1 was originally identified in the nervous system. Recent studies have shown its expression in dendritic cells, macrophages (alveolar, bronchial, and intravascular; tumor-associated macrophages), T cell subpopulations (CD8+ T cells, Treg cells, Tfh cells, and NKT cells), and mast cells (Bruder et al. 2004; Chekol Abebe et al. 2021; Marone et al. 2016; Roy et al. 2017; Tordjman et al. 2002) demonstrating the important role of NRP1 in the regulation of immune response and in respiratory diseases. NRP1 was also found to be expressed on osteoblasts, renal glomerular mesangial cells, glomerular epithelial cells, neuroendocrine cells of the gastrointestinal tract, adipocytes, kidney’s podocytes, olfactory epithelium, and olfactory neurons (reviewed in Chekol Abebe et al. 2021; Roy et al. 2017; Ellis 2006). Mouse NRP1 is a critical receptor for Sema4A acting on Treg cells to regulate their stability and function (Delgoffe et al. 2013). In humans, Plexin B1 plays this Sema4A-dependent potentiating function for Treg cells which lack NRP1 expression (Chapoval et al. 2019). NRP1 is also expressed on CD4^low^ PBMC-derived human monocytes (Chapoval et al. 2019). Therefore, these monocytes might potentially get infected with SARS-CoV-2 virus although the ACE-2 expression on these cells has not been assessed. Interestingly, NRP1 expression was previously detected on lung tissue macrophages by IHC (Aung et al. 2016). These macrophages are believed to be a major source of cytokine overexpression in the “cytokine storm” phenomenon observed in severe SARS-CoV-2 infection (Merad and Martin 2020).

Peptides with a C-terminal basic sequence motif (C-end rule or CendR motif) bind to NRP1 and are taken into cells by endocytosis (Teesalu et al. 2009). The binding of the S1 CendR motif generated by the furin cleavage of Spike protein to NRP1 did not affect cell surface attachment but promoted cell entry and infection by SARS-CoV-2 (Daly et al. 2020). As NRP1is known to mediate the internalization of CendR ligands through an endocytic process resembling micropinocytosis (Teesalu et al. 2009), it is possible that S1 interaction with NRP1 alone, even in the absence of ACE2, may induce a complex internalization presumably by the receptor-mediated CendR endocytosis. This pathway was previously demonstrated in vitro using the NRP1 plasmid-transfected HeLa cells (Pang et al. 2014). However, the intracellular consequences of such internalization for human primary cells have not been explored.

## Host immune response to SARS-CoV-2

**Fig. 2:**
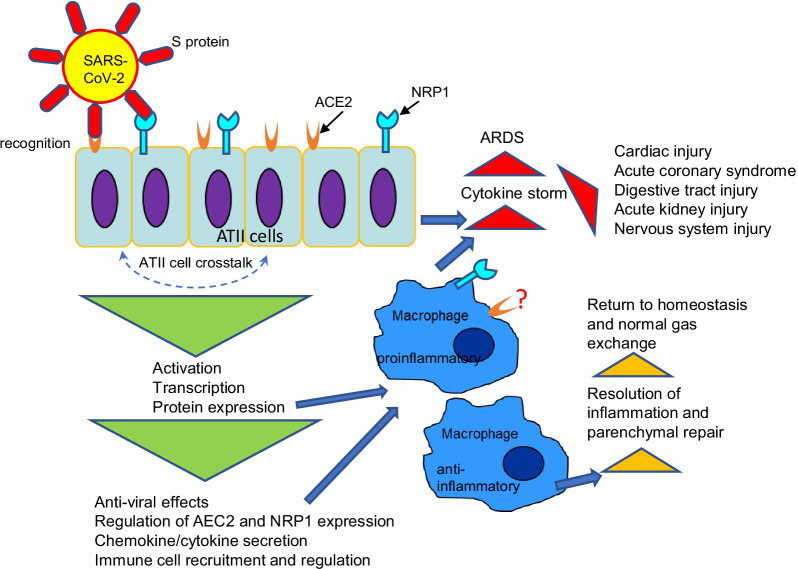
Integrative model of ATII cell contribution to alveolar macrophage activation and cytokine storm in SARS-CoV-2 infection. ATII cells directly interact with pathogen utilizing ACE2 and NRP1. They induce specific signature signals which activate sentinel cells in the airways to further mount a response. These sentinel cells can reciprocally activate ATII cells by secretion of proinflammatory cytokines. In severe form of disease (red triangles), activation of these cells leads to hyperproduction of cytokines and lung failure. In mild form of infection (orange triangles), anti-inflammatory signature is evoked which eventually leads to macrophage directed initiation of resolution of inflammation and parenchymal repair. Phenotype of alveolar macrophages in SARS depends on a delicate balance between negative and positive regulatory pathways activated by ATII cells. *ARDS* acute respiratory distress syndrome

Host immune response plays a critical role in protection and fight against SARS-CoV-2. SARS-CoV-2, primarily distributed by air droplets, infects the host’s respiratory system. Multiple recent studies have shown that patients infected with SARS-CoV-2 demonstrated pathologic changes in multiple tissues and organs including the gastrointestinal and pancreaticobiliary systems, kidney, heart, and central nervous system (Cheng et al. 2020; Inamdar et al. 2020; Mao et al. 2020; Huang et al. 2021; Karras et al. 2021; Raman et al. 2021; Song et al. 2021a, b). It has been reported that many patients with severe disease have an exaggerated immune response to virus with elevated levels of proinflammatory cytokines IL-1, IL-6, IL-12, and increased IFNγ, IFNγ-inducible protein 10, IL-8 (neutrophil chemoattractant), and MCP-1 (monocyte chemoattractant protein 1) (Crestani et al. 1994; Wong et al. 2004a; Zhang et al. 2004). Within the lung tissue, ATII cells could serve as a source of those cytokines and chemokines as they were reported to make IL-6 in vitro and in vivo and participate in intra-alveolar cytokine networks secreting IL-8, IFN, MCP-1, TGFβ, and GM-CSF (Fig. 2) (Crestani et al. 1994; Lin et al. 1998; Yan et al. 2018). Therefore, ATII cells may contribute to a local inflammatory response in viral infection by producing chemokines which attract inflammatory cell sequestration into the sites of infection and cytokines which activate those immigrant and local cells (Fig. 2). One recent study in SARS-CoV-2 patients have shown that in addition to the cytokines and chemokines listed above, TNFα, IL-2, and IL-7 were significantly elevated in a subset of patients with a fulminant and fatal hypercytokinaemia (Mehta et al. 2020).

## Neurologic consequences of SARS-CoV-2 infection and role of NRP1

SARS-CoV-2 directly and indirectly interacts with the peripheral nervous system and causes pain (McFarland et al. 2021). SARS-CoV-2 induces a complex neurovirulence in humans, a consequence of virus interaction with dorsal root ganglia and trigeminal ganglia. There are distinct neurological effects of SARS-CoV-2 infection on the early and late stages of disease. The human body respond to SARS-CoV-2 by the production and release of type I interferons (McFarland et al. 2021). These cytokines directly interact with corresponding receptor complexes expressed on nociceptors and thus promote pain. This mechanism of SARS-CoV-2 action explains headaches and body aches associated with the disease. However, some patients with severe COVID-19 infection lack type I interferon involvement due to selected SARS-CoV-2 proteins with antagonistic action against IFN-signaling pathways. Another subset of patients which also lack type I IFN involvement because of internal secretion of auto-Abs against type I INFs was particularly susceptible to severe disease (Bastard et al. 2020; McFarland et al. 2021; Lopez et al. 2021; Zhang et al. 2020). Additional cytokine-related mechanism of body aches and pain in SARS-CoV-2 infection is directly linked to ACE2 expression on human skeletal muscles which leads to muscle tissue damage during infection, tissue inflammation, and elevated levels of IL-6 which is known for its nociceptive effects. The inflammatory neuronal demyelination resembling Gullian-Barre syndrome was observed in a subset of patients with severe infection could lead to chronic muscle weakness and fatigue (Finsterer et al. 2021). Dorsal root ganglia are reported to express ACE2, furin, and NRP1, the complete receptor machinery for SASR-CoV-2 infection. Interestingly, SARS-CoV-2 interaction with NRP1 through its Spike protein binding on nociceptors causes the pain-lowering effects in contrast to its pain-promoting interaction with ACE-2 (Finsterer et al. 2021). This NRP1-linked phenomenon might explain the increased disease transmission in asymptomatic infected individuals (Finsterer et al. 2021; Karuppan et al. 2021). Studies in human brain organoids have clearly demonstrated the SARS-CoV-2 neurotropism but these studies focused on ACE-2 expression and function and omitted the significance of NRP1 (Jacob et al. 2020; Ramani et al. 2021).

Viral infections cause damage to the nervous system with clinical features varying from encephalitis to meningitis with various degrees of severity (Big et al. 2009; Karuppan et al. Karuppan et al., 2021; Singh et al. 2021). SARS-CoV-2 invasion of central nervous systems caused many pathologic effects ranging from reversible brain dysfunction syndrome with headache, dysphoria, mental disorders, and delirium (Mao et al. 2020) to more sever pathologies such as edema of the brain tissue and partial neuronal degeneration (Dixon et al. 2020). The contributions of ACE2 and co-receptor NRP1 to these processes were the subjects of several recent studies (Davies et al. 2020; Karuppan et al. 2021; Khan and Gomes 2020). Whereas ACE-2 expression is rather low in human lung tissue and olfactory epithelium, NRP1 was found to be highly expressed in the lungs and olfactory tubercles and paraolfactory gyri of the human brain (Davies et al. 2020; Karuppan et al. 2021). The detailed examination of a precise NRP1 expression in human brain pointed to a hippocampal formation as the highest NRP1 expressor whereas on the cellular level its expression was detected in endothelial cells, mural cells, neuron clusters, perivascular macrophages and microglia (Davies et al. 2020). The SARS-coV-2 interaction with NRP1 could explain the observed virus entry into olfactory epithelial cells and loss of olfactory function, the ability to detect odorous molecules (sense of smell) in COVID-19 disease. The reduced pain perception in infected asymptomatic patients could be, in part, related to the effect of Spike protein blocking nociceptic VEGF-A/NRP1 interaction (Moutal et al. 2021). SARS-CoV-2 genome was detected in cerebrospinal fluids or infected people and selected patients showed the presence of viral encephalitis (Karuppan et al. 2021). The documented brain dysfunction problems in SARS-CoV-2 infection suggested that the virus can cause infectious toxic or even acute necrotizing encephalopathy. In some patients the virus can lead to the formation of newly detected demyelinating lesions in the brain. All these reports together with known critical function of NRP1 in brain development including the neuronal axon guidance, target recognition, and synaptogenesis (Telley et al. 2016) point to an important role of NRP1 as the Spike protein receptor in the development of serious and prolonged neurological aspects of SARS-CoV-2 infection.

## ACE2 and NRP1 targeting strategies to combat SARS-CoV-2 infection

Several therapeutic strategies for SARS-CoV-2 infection have been proposed, most of them targeting the SARS-CoV-2 Spike–ACE2 interaction (Chan et al. 2020; Kruse 2020; Montail et al. 2020; Wong et al. 2004b). They include: (1) small SARS-CoV-2 Spike protein molecule of 193 aa long containing ACE2-binding domain (RBD) which effectively blocked the virus entry in cell cultures; (2) anti-ACE2 blocking Ab; and (3) soluble ACE2-Fc fusion protein (Lei et al. 2020) to bind and neutralize S protein (Table 1). The recently published review by Xiaojie et al. (2020) details the Spike protein targeting drugs based on ACE2 sequences. These drugs include: (1) recombinant soluble ACE2 ectodomain, and (2) its selective mutants which either bind Spike with a higher affinity or express a low catalytic activity which preserve ACE2 function in physiological processes (Table 1). The article by Xiaojie et al. (2020) also summarized the recent development and major structural characteristics of SARS-CoV-2 neutralizing mAbs isolated from convalescent patients, immunized animals, and phage-displayed human antibody libraries. Of note, all these mAbs currently in several clinical studies at different biotech companies were found to target S1 protein and none of them target S2 protein.

**Table 1:** A summary table on SARS-CoV-2 infection targeting strategies

	Research	References
Targeting Spike–ACE2 interaction		
Small SARS-CoV-2 Spike protein molecule of 193 aa long containing ACE2-binding domain (RBD)	Effectively blocked the virus entry in cell cultures	Several studies reviewed in Kruse (2020)
Anti-ACE2 blocking Ab	Immunization of animals and Ab library screening Human Ab library screening	Reviewed in Kruse (2020), Xiaojie et al. (2020)
Soluble ACE2-Fc fusion protein to bind and neutralize S protein	Extracellular domain of human ACE2 fused with the Fc region of the human immunoglobulin IgG1 shows high-affinity binding to the RBD of SARS-CoV-2 and potent neuralization of virus entry in vitro in cell lines	Kruse (2020), Lei et al. (2020)
Recombinant soluble ACE2 ectodomain	Effectively competes with native ACE2 on cell surface to block the subsequent fusion steps. Inhibit growth of authentic SARS-CoV-2 in Vero cells and in human organoids	Xiaojie et al. (2020), Monteil et al. (2020)
Recombinant ACE2 mutants	Either bind Spike with a higher affinity or express a low catalytic activity which preserve ACE2 function in physiological processes	Xiaojie et al. (2020), Monteil et al. (2020), Chan et al. (2020)
Anti-Spike Ab from convalescent plasma	mAbs from a COVID-19 infected subject 21 days after the onset of disease identified by using Spike protein as a bait	Several studies reviewed in Kruse (2020), Lei et al. (2020)
Bi-specific fusion protein, ACE-MAB	One arm is human high affinity anti-Spike Ab. The other arm is a truncated ACE2 protein that binds to a different epitope of Spike	Sorrento therapeutics (reviewed in Xiaojie et al. 2020)
Targeting Spike–NRP1 interaction		
Anti-NRP1 neutralizing Ab	Incubation of Caco-2 cells with anti-NRP1 neutralizing mAbs reduced SARS-CoV-2 infection compared to a control mAb targeting avian influenza A virus (H11N3) hemagglutinin	Cantuti-Castelvetri et al. (2020), Daly et al. (2020)
Small molecule EG00229 acting as a selective NRP1 antagonist	Binds the b1 CendR binding pocket and inhibits VEGF-A binding by NRP1. Incubation of Caco-2 cells with EG00229 reduced the efficiency of SARS-CoV-2 infection	Daly et al. (2020)
Soluble b1b2 domain of NRP1	When SARS-CoV-2 pseudovirus was preincubated with recombinant, soluble extracellular b1b2 domain of NRP1, the wild type significantly reduced cell infection	Cantuti-Castelvetri et al. (2020)
Small molecule inhibitors for Spike–NRP1 interaction	Approach similar to the one recently used to identify six compounds which effectively disrupted VEGF-A–NRP-1 interaction	Perez-Miller et al. (2020)

**Fig. 3:**
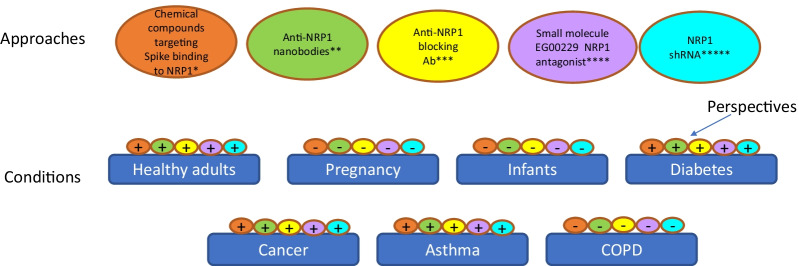
Potential approaches to targeting NRP1 for the treatment of SARS-CoV-2 infection in patients with or without comorbidities. Expressed in temporary restricted manner in the embryo, NRP1 is important for cardiovascular and pulmonary system development. NRP1 expression was not detected in healthy pancreas or in pancreatitis but was upregulated in pancreatic cancer. Most evidence point to tissue-damaging role of NRP1 in all types of cancer. NRP1 ligand VEGF_165_ plays critical role in asthma whereas NRP1 is protective in COPD pathobiology

Three NRP1 targeting strategies were used to define its critical role in SARS-CoV-2 infection in vitro, namely: (1) anti-NRP1 neutralizing Ab (Cantuti-Castelvetri et al. 2020; Daly et al. 2020); (2) the small molecule EG00229 acting as a selective NRP1 antagonist (Daly et al. 2020), and (3) soluble extracellular b1b2 domains of NRP1 (Cantuti-Castelvetri et al. 2020) (Table 1). Incubation of Caco-2 cells with anti-NRP1 mAbs raised against the b1b2 ectodomain of NRP1 significantly (by ~ 40%) reduced SARS-CoV-2 infection as compared to a control mAb targeting avian influenza A virus (H11N3) hemagglutinin (Cantuti-Castelvetri et al. 2020; Daly et al. 2020). Other targeting approach of SARS-CoV-2 entry into cells was based on b1/b2 module of NRP1 as a binding site for VEGF_165_ (Perez-Miller et al. 2020). A library of 5 × 10^5^ compounds was screened for specifically targeting this site; nine chemical series were reported with lead- or drug-like physical and chemical properties, out of which six compounds effectively disrupted VEGF-A–NRP-1 interaction, and all nine inhibited VEGF-A triggered VEGFR2 phosphorylation. These series of lead compounds represent a first step in development of small molecule inhibitors for the SARS-CoV-2 S–NRP1 interaction (Fig. 3) and for certain types of cancer where NRP1 plays a disease-promoting role.

## NRP1 in immune response and its targeting in SARS-CoV-2 infection in adults without comorbidities

The important roles of NRP1 in the immune response have been discussed in several published reviews (Chuckran et al. 2020; Roy et al. 2017; Ellis 2006; Chaudhary et al. 2014; Li et al. 2016). All NRP1-expressing cells may be potential targets for SARS-CoV-2 because Spike protein through its CendR motif that is exposed following furin processing interacts with NRP1 that can leads to a complex internalization through previously described endocytosis (Daly et al. 2020; Teesalu et al. 2009; Jobe and Vijayan 2021). Of note, recent reports clearly showed that without ACE2, NRP1 alone is not able to support efficient virus infection, not even in the immunocompromised HEK 293T cell system (Cantuti-Castelvetri et al. 2020; Daly et al. 2020). Moreover, NRP1 depletion did not affect virus binding to the cell surface but specifically affected the virus uptake (Daly et al. 2020). It is still debated whether SARS-CoV-2 might infect NRP1-expressing DC. However, several previous studies have shown that MERS-CoV and SARS-CoV use DC-SIGN as an alternative receptor for DC infection or as a receptor which facilitates virus infectivity through ACE2 (Yang et al. 2004; Marzi et al. 2004; Campana et al. 2020). DC are unmatched in their ability to prime naïve CD4+ T cells and initiate and propagate the immune response to given antigen. Among immune cells, NRP1 expression was first identified on human plasmacytoid dendritic cells (pDCs) (Dzionek et al. 2002). Recent findings detect a significant drop in circulating pDC number in SARS-CoV-2 infected patients (Zhou et al. 2020). The mechanisms of such SARS-CoV-2 effect on pDC were not defined. pDCs are known for their type I IFN secretion in response to viral infection. Human pDCs showed potent IFNa release in response to Respiratory Syncytial Virus (RSV) which was inhibited by anti-NRP1 Ab pretreatment of cells (Grage-Griebenow et al. 2007). In addition to its expression on pDCs, NRP1 was also detected on conventional DCs isolated from human peripheral blood, where it potentially promotes T cell priming by mediating the formation of immunological synapse between cells (Tordjman et al. 2002). NRP1 is expressed on immature DC (iDCs). iDCs do not express costimulatory molecules, are not activated, and can not stimulated T effector cells. They induce T cell anergy and T cell deletion instead. However, they can stimulate Treg cells to secrete immunosuppressive cytokines such as IL-10 and TGF-β (Mahnke et al. 2002). NRP1 was defined as a ‘glue’ between Tregs and DCs (Mizui and Kikutani 2008; Song et al. 2020). Thus, NRP1 is a critical molecule for immune response to foreign antigen. At the same time, SARS-CoV-2 virus could use NRP1 for DC invasion. It is unclear if DC co-express ACE2 or upregulate ACE2 expression after Spike binding to NRP1. One current study reported little to no expression of ACE2 on immune cells in human peripheral blood including B cells, NK cells, monocytes, dendritic cells, granulocytes, and all subtypes of T cells (Song et al. 2020). However, ACE2 expression was induced or upregulated on CD3+ T cells obtained from infected patients while CD20+ B cells and the CD16+/HLA-DR+ monocytic/dendritic cells did not change a relatively low ACE2 level as compared to same cell populations obtained from healthy volunteers (Osman et al. 2020).

One published study reported the absence of any significant effect of anti-NRP1 Ab raised to VEGF-binding site of NRP1 on HUVEC cell proliferation and permeability (Pan et al. 2007). This Ab also did not affect VEGF-induced phosphorylation of Erk1/2 or Akt in endothelial cells but significantly inhibited VEGF-induced cell migration. There is one published report on the phase I study of anti-NRP1 mAb in patients with advanced solid tumors where this was a well-tolerated approach with promising results (Weekes et al. 2014). Jung et al. (2020) used the NRP1 antagonist [Fc(AAG)-TPP11] generated by fusion of the NRP1-specific binding peptide TPP11 with the C-terminus of an effector function-deficient immunoglobulin Fc(AAG) variant to inhibits intratumoral NRP1+ Treg cell function and stability. The intraperitoneal injections of Fc(AAG)-TPP11 into mice with established tumors demonstrated a potent anti-tumoral effect and inhibited tumor growth by > 70%. The NRP1 antagonist effect was comparable to that observed with the use of Treg-depleting anti-CTLA-4 Ab. Other NRP1-directed drugs or biologics have never been examined in vivo.

## Perspectives of targeting NRP1 in SARS-CoV-2 infection in pregnant women and infants

NRP1 is required for normal embryonic vascular, cardiac, and limb development (Kitsukawa et al. 1995; Kawasaki et al. 1999). It was reported to be expressed in a temporary restricted manner in the mouse embryo suggesting that its ectopic or excess expression could cause abnormalities (Kitsukawa et al. 1995). Indeed, chimeric embryos with NRP1 overexpression showed abnormal body vascularization and severe pathologies to the cardiovascular and nervous tissues. Such embryos demonstrated lethality at maximum 17.5 pdc (post-conception days of embryonic age) as the result of profound abnormalities to both systems. Therefore, the exogenous NRP1 overexpression causes embryonic death. Such effect was attributed to the unique NRP1 structure which has three domains all potentially involved in different molecular interactions. The observation that different systems are affected by NRP1 overexpression also suggests that NRP1 is a multifunctional molecule. The authors concluded that the correct spatiotemporal patterns of NRP expression were essential for the growth and fasciculation of nerve fibers, formation of the cardiovascular system, and for the limb’s formation. Interestingly, a targeting disruption of nrp1 gene was also reported to be embryonic lethal and resulted in multiple defects to the cardiovascular system (Kawasaki et al. 1999). Therefore, NRP1 targeting with Abs or small molecule inhibitors may lead to adverse events in SARS-CoV-2 infection in pregnant women and infants (Fig. 3). However, the drugs based on NRP1 sequence/structure which selectively bind and neutralize SARS-CoV-2 spike protein could be potentially very useful in these groups.

## Targeting strategies for NRP1: cancer models

A number of NRP1-targeting strategies have been developed to treat cancer. The overexpression of NRP1 is reported in several types of cancers such as colon (Parikh et al. 2004), stomach (Mei et al. 2020), lung (Kawakami et al. 2002; Lantuejoul et al. 2003), osteosarcoma (Handa et al. 2000), breast (Stephenson et al. 2002), astrocytoma (Broholm and Laursen 2004), glioma (Hu et al. 2007), melanoma (Graziani and Lacal 2015; Bao et al. 2021), and is considered to be a negative prognostic marker for the disease outcome (reviewed in Ellis 2006; Rizzolio and Tamagnone 2011; Wild et al. 2012). Elevated NRP1 expression was detected in malignant cells and in tumor microenvironment (TME) on macrophages, Treg cells, CD8+ T cells, and dendritic cells (DCs) (reviewed in Chuckran et al. 2020; Moutal et al. 2021; De Vlaemnick et al. 2020; Liu et al. 2020). All these together established a strong association between NRP1 and cancer. Several NRP1-directed therapies such as anti-NRP1 mAb, anti-NRP1 nanobodies, and NRP1 antagonist Fc(AAG)-TPP11 demonstrated significant retardation of established tumors without noticeable toxicity (Wild et al. 2012; Weekes et al. 2014; De Vlaeminck et al. 2020; Jung et al. 2020). These studies demonstrate that NRP1 targeting can be highly effective with minimal adverse effects. It could also be considered as an approach in managing and treating the severe SARS-CoV-2 infection in cancer patients (Fig. 3).

## Targeting NRP1 in SARS-CoV-2 infection in patients with pulmonary diseases

Two research groups have described abundant expression of NRP1 in alveolar epithelium (Ito et al. 2000; Roche et al. 2002). However, the functional significance of such expression is unknown. NRP1 expression on epithelial cells may modulate the balance between VEGF and Sema3 signaling in nonepithelial cells (Bagnard et al. 2001; Castro-Rivera et al. 2004). Local lung NRP1 levels gradually increase in the process of lung organogenesis (Roche et al. 2002). NRP1 and its ligands Sema3A and VEGF contribute to the process of alveolar septation aimed to increase blood-gas interchange to supply oxygen to developing organism (Ito et al. 2000; Gerber et al. 1999; Jakkula et al. 2000; McGrath-Morrow et al. 2005; Kunig et al. 2005). Sema3A inhibits branching morphogenesis in lung bud organ cultures acting via NRP1 (Roche et al. 2002), while Sema3C and Sema3F promote lung branching morphogenesis using both NRP1 and NRP2 (Kagoshima and Ito 2001). The NRP1 expression was downregulated in smokers with diagnosed cigarette smoking-induced COPD which displayed defective lung function when compared with smokers with normal lung function and nonsmoking control subjects (Marwick et al. 2006). Therefore, NRP1 plays a protective role in COPD pathobiology and direct targeting of NRP1 in patients with COPD may not be beneficial in case of SARS-CoV-2 infection. Studies in murine models have demonstrated a critical role of NRP1 in proper structural maintenance of lung alveoli (Le et al. 2009). There were more disruptions to alveolar structure in chronic cigarette smoke exposure in mice with conditional epithelial Nrp1 deletion compared to similarly treated WT mice. Interestingly, bronchoalveolar lavage cell composition did not differ significantly between two experimental mouse groups.

The expression of NRP and its ligands in lung cancer is widely reported. Overexpression of both, NRP1 and NRP2 was reported in non-small cell lung carcinoma and it significantly correlated with tumor progression (Kawakami et al. 2002). High levels of NRP1 expression correlated with shorter disease-free and overall survival, and combined overexpression of NRP1 and NRP2 was associated with a worse prognosis than when either NRP was singly overexpressed (Kawakami et al. 2002). A progressive upregulation of NRP levels was observed in different types of lung cancers including squamous cell carcinoma, small cell lung carcinoma, adenocarcinoma, large cell neuroendocrine carcinoma, basaloid carcinoma, and typical and atypical carcinoids (Lantuejoul et al. 2003). Expression progresses starting from benign bronchial hyperplasia to dysplasia and then to invasive carcinoma and was correlated with increases in VEGF expression (Lantuejoul et al. 2003). VEGF_165_, one of NRP1 ligands, plays a critical role in allergic asthma (Lee et al. 2004; Bhandari et al. 2006; Chapoval et al. 2009). Expression of both, NRP1 and its another ligand, Sema3A, was found to be upregulated in sputum of asthmatic patients and in BAL and lung homogenates of mice with OVA-induced experimental asthma (Shim et al. 2013) suggesting NRP1 and its ligands play a pathologic role in allergic asthma (Fig. 3). It is possible that targeting of NRP1 to combat SARS-CoV-2 infection in lung cancer and asthma patients will reduce or prevent the severe form of infection.

## Targeting NRP1 in SARS-CoV-2 infection in patients with diabetes

The rate of SARS-CoV-2 infection in people with diabetes is similar to that of general population but post-infection complications are different and much more severe (Apicella et al. 2020; Feldman et al. 2020; Zhou et al. 2021). Moreover, the prevalence of diabetes was 34.6% in patients with severe COVID-19 (Apicella et al. 2020). One recent report shows that transmembrane protein endothelial and smooth muscle cell-derived neuropilin-like protein (ESDN) serves as an inhibitor of insulin receptor signal transduction in the liver, muscle, and adipose tissue (Li et al. 2016). However, the mechanisms of ESDN action in angiogenesis are distinct from those defined for NRP1 (Nie et al. 2013). A recent study by Wu et al. (2021) found that b-cells in human pancreas express ACE2, TMPRSS2, and selectively high NRP1. SARS-CoV-2 infection in patients affected pancreatic insulin levels, its secretion, and induces b-cell apoptosis. All these events were rescued by NRP1 inhibition. Therefore, b-cells display the necessary molecular machinery for efficient SARS-CoV-2 infection and high NRP1 expression may, in part, explain the SARS-CoV-2 tropism for b-cells. Indeed, a comprehensive analysis of human pancreatic tissue employing the immunofluorescence, immunohistochemistry, RNA scope and electron microscopy demonstrated that 70% of ACE2 expression was found on pancreas vasculature, whereas b-cell are ACE2+ only in 30% (Steenblock et al. 2021). The high NRP1 expression on b-cells suggested that the virus uptake could be facilitated by NRP1 when ACE2 expression is low. Therefore, it is tempting to speculate that specific targeting of NRP1 in severe SARS-CoV-2 infection may be a potentially useful strategy in patients with diabetes (Fig. 3).

## Other receptors involved in SARS-CoV-2 infection

Other receptors can facilitate SARS-CoV-2 entry into cells. One such receptor is CD147 or basigin, a transmembrane glycoprotein of the immunoglobulin superfamily which has been shown to increase infectivity of both SARS-CoV and SARS-CoV-2 viruses (Chen et al. 2005; Wang et al. 2020c). The study by Wang et al. (2020c) demonstrated a co-localization of CD147, Spike protein, and Rab5 in BHK-21 cells induced to express CD147 and in lung tissues from patient with SARS-CoV-2 infection. This observation let the authors to conclude that Spike protein binding to CD147 induced a CD147-mediated endocytosis of virus. The results of this study also showed the infection of lung T cells obtained from COVID-19 patients which was mediated by CD147 but not by ACE2 as T cells lack ACE2 expression. The cell infectivity was inhibited by a humanized IgG2 antibody targeting CD147, meplazumab, in a dose-dependent manner. Moreover, the constructed hCD147 transgenic mice, unlike WT littermates, were susceptible to SARS-CoV-2 infection and developed virus-induced pneumonia what further establishes CD147 as an alternative receptor for SARS-CoV-2 entry in an ACE2-deficient environment. However, a recent report by Shilts et al. (2021) showed no evidence for a direct interaction of the SARS-CoV-2 spike protein with basigin. HEK293 cells transfected with basigin full-length cDNA overexpression plasmid were not able to bind a full-length Spike or its S1 domain. Moreover, CRISPR-Cas9-mediated CD147 knockdown in a lung cell line had no effect on cell’s susceptibility to SARS-CoV-2 infection whereas knockdown of ACE2 completely blocked viral infection in CaLu-3 cells. Based on these new data, the authors suggested to re-consider all hypotheses relying on Spike protein binding CD147 to explain viral tropism but rather focus on indirect CD147 involvement in SARS-CoV-2-induced disease.

Several recent studies established apilimod, a known pharmacologic blocker of PIKfyve (a 240 kDa class III lipid PI-3P-5-kinase present on the cytosolic face of endosomal membranes) as a potent anti-SARS-CoV-2 agent (Baranov et al. 2020; Kang et al. 2020; Kreutzberger et al. 2021; Riva et al. 2020). Apilimod inhibits the viral capsid fusion with the host cell membrane by inhibiting different lysosomal proteases such as TMPRSS2, furin, trypsin, matriptase, and cathepsin B/S/L (Baranov et al. 2020).

To study a potential interaction between Spike protein and distinct TLRs, the 3D structures of Spike and TLR1, TLR2, TLR4, and TLR5 were retrieved from the RCSB Protein Data Bank and used in the in silico molecular docking studies (Choudhury and Mukherjee 2020). Significant binding of SARS‐CoV‐2 Spike protein to TLR1, TLR4, and TLR6 was detected with a respective binding energy value of − 57.3, − 120.2, and − 68.4. Interestingly, the strongest interaction of Spike protein was with TLR4. This finding was further supported by the in vitro studies where a direct Spike-TLR4 interaction was demonstrated employing TLR4-expressing THP1 cells. Spike protein induced IL-1b production in these cells in a dose-, NF-kB-, and MyD88-dependent manner which was inhibited by the TLR4-specific blocker Resatorvid. MD2 and CD14 were also involved in TLR4 activation by Spike as demonstrated by use of specific blockers such as T5342126 and anti-CD14 Ab, respectively.

Therefore, several immune and non-immune receptors can potentially be utilized by SARS-CoV-2 Spike protein to infect different cells. Such Spike-receptor association may play either a protective or a damaging role in virus-induced disease, an area of research that needs to be further investigated and clarified.

## Conclusions

NRP1 has been shown to participate in the entry of SARS-CoV-2 into cells. Importantly, recent ‘omic’ analyses revealed a significant upregulation of NRP-1 in biological samples from COVID-19 patients compared to healthy controls (Xiong et al. 2020). Moreover, the increased levels of one NRP1 ligand, VEGF_165_, in bronchial alveolar lavage fluid from COVID-19 patients were also reported (Xiong et al. 2020). VEGF_165_ is a physiological ligand for the b1b2 pocket in NRP-1 (Moutal et al. 2021). Spike protein, the major surface antigen of SARS-CoV-2, could block VEGF_165_/NRP-1 signaling. Noteworthy, Sema3A and Sema4A also bind NRP-1 but into a1a2 pocket (Fig. 1). Nevertheless, Sema3A or Sema4A binding may modify the ability of NRP-1 to interact with Spike. The multiple roles of these ligand-receptor interactions in SARS-CoV-2 infection of different cells, tissues, and organs need to be further evaluated. This is an emerging field in research as the roles of NRP1 in different diseases are not well established. Nevertheless, published studies point to NRP1 as potential player in SARS-CoV-2 infection and recently developed approaches for NRP1 targeting provide a foundation for designing novel anti-viral therapies.

## Data Availability

The dataset supporting the conclusions of this article is included within the article.

## Abbreviations

ACE2: Angiotensin converting enzyme 2
NRP-1: Neuropilin-1
SARS-CoV-2: Severe acute respiratory syndrome coronavirus 2
COVID-19: Coronavirus disease 2019
VEGF_165_: Vascular endothelial growth factor-A
EU CHFS: European Union Commission for Health and Food Safety
MasR: G-protein coupled Mas receptor
Akt: Protein kinase B
NOS: Nitric oxide species
Sema3A: Semaphorin 3A
Sema4A: Semaphorin 4A
ATII: Alveolar epithelial type II cells
IHC: Immunohistochemistry
CendR: C-end rule
PPC1: Human prostatic cell line
HUVEC: Human umbilical vein endothelial cells
RBD: Receptor-biding domain
RSV: Respiratory syncytial virus
iDC: Immature DC
OVA: Ovalbumin
